# Valvular Heart Disease following Anthracycline Therapy—Is It Time to Look beyond Ejection Fraction?

**DOI:** 10.3390/life12081275

**Published:** 2022-08-20

**Authors:** David Zahler, Joshua H. Arnold, Tali Bar-On, Ari Raphael, Shafik Khoury, Zach Rozenbaum, Shmuel Banai, Yaron Arbel, Yan Topilsky, Michal Laufer-Perl

**Affiliations:** 1 Department of Cardiology, Tel-Aviv Sourasky Medical Center, Tel Aviv 6423906, Israel; 2 Sackler School of Medicine, Tel Aviv University, Tel Aviv 6997801, Israel; 3 Department of Medicine, University of Illinois at Chicago, Chicago, IL 60607, USA; 4 Internal Medicine T, Tel-Aviv Sourasky Medical Center, Tel Aviv 6423906, Israel; 5 Department of Oncology, Tel-Aviv Sourasky Medical Center, Tel Aviv 6423906, Israel

**Keywords:** echocardiography, cardio-oncology, cardiotoxicity, valve, mitral regurgitation, tricuspid regurgitation

## Abstract

The association between anthracycline (ANT) and left ventricle (LV) dysfunction is well known; however, data regarding its direct effect on cardiac valve function is limited. We aimed to evaluate how ANT therapy affected valvular function in patients diagnosed with breast cancer. Data were prospectively collected as part of the Israel Cardio-Oncology Registry (ICOR). Patients underwent echocardiography exams at baseline (T1), during ANT therapy (T2), and after completion within 3 months (T3) and 6 months (T4). A total of 141 female patients were included, with a mean age of 51 ± 12 years. From T1 to T4, we observed a significant deterioration in LV ejection fraction (60.2 ± 1.5 to 59.2 ± 2.7%, *p* = 0.0004) and LV global longitudinal strain (−21.6 (−20.0–−23.0) to −20.0 (−19.1–−21.1)%, *p* < 0.0001)), and an increase in LV end-systolic diameter (25 (22–27) to 27 (24–30) mm, *p* < 0.0001). We observed a significant increase in the incidence of new mitral regurgitation (MR) development (4 to 19%, *p* < 0.0001), worsening with concomitant trastuzumab therapy (6% to 31%, *p* = 0.003), and a trend for tricuspid regurgitation development (4% to 8%, *p* = 0.19). ANT therapy is associated with the development of a new valvular disease, mainly MR, which may imply the need for a valvular focus in the monitoring of cancer patients.

## 1. Introduction

Advancements in early detection and cancer therapies have led to a significant improvement in disease-free years and overall survival for patients diagnosed with breast cancer [1]. This extended lifespan has revealed long-term adverse sequelae that are experienced by survivors, especially the development of clinically significant cardiotoxicity [2,3,4]. While anthracycline (ANT) therapy, specifically with doxorubicin, is considered to be the most notorious drug to induce cardiotoxicity [3], it is still the foundation of protocolized therapy for breast cancer [5].

To date, two-dimensional (2D) echocardiography has been used to monitor and diagnose cardiotoxicity during cancer therapy through changes in left ventricular ejection fraction (LVEF) [3,6,7]. While the association between LVEF reduction and cardiac outcomes and mortality was well documented [8], LVEF evaluation is less sensitive to early minor myocardial damage [9,10]. Furthermore, changes in LVEF are detectable only in the presence of large myocardial tissue damage that is likely to be irreversible [11,12]. Lately, the use of 2D speckle tracking echocardiography (2D-STE), specifically global longitudinal strain (GLS), is considered the optimal tool for evaluating cancer patients’ heart function due to its ability to detect subclinical myocardial changes [3,6,7,13].

While the effect of ANT on LVEF and LVGLS is well known, data regarding the direct effect of ANT on cardiac valve function is limited [3]. Longitudinal studies following patients treated early in life for Hodgkin’s lymphoma showed a significant risk for the development of valvular heart disease, but this was seen mainly in those who received direct mediastinal radiotherapy [14]. While many of these patients were treated with ANT therapy, data regarding those treated solely with chemotherapy and its effects on valvular heart disease is lacking.

The aim of our study was to evaluate the direct effect of ANT therapy on valvular function in patients diagnosed with breast cancer.

## 2. Materials and Methods

### 2.1. Study Population

This study was part of the Israel Cardio-Oncology Registry (ICOR) [13,14,15,16,17,18,19,20], which enrolls patients evaluated in the cardio-oncology clinic at Tel Aviv Sourasky Medical Center. All patients signed an informed consent form during their first visit to the clinic and were then followed prospectively. The registry was approved by the local ethics committee (Tel Aviv Sourasky medical center ethic committee, identifier: 0228-16-TLV) and is registered on clinicaltrials.gov (identifier: NCT02818517). The inclusion criteria for this cohort were female patients diagnosed with breast cancer and treated with doxorubicin therapy with a cumulative dose of doxorubicin ≥180 mg/m^2^, which is considered to be cardiotoxic [21]. According to our facility surveillance protocol, all patients treated with ANT underwent a follow-up evaluation at the following time points: at baseline, before ANT exposure (T1), during doxorubicin therapy after a cumulative dose of 180–240 mg/m^2^ (T2), and following the completion of doxorubicin therapy within 3 months (T3) and 6 months (T4). Exclusion criteria included aged below 18, past ANT therapy, dexrazoxane (cardioxane) therapy, and baseline LVEF < 55%.

### 2.2. Study Protocol

A complete baseline medical history, cardiac risk factors, and medical treatment were noted in all patients from the electronic medical charts. Echocardiographic routine parameters, including LVEF, LVGLS, diastolic function, valvular function, and right ventricular (RV) function, were evaluated, as described in the echocardiography section. Cardiotoxicity was defined according to the ESC 2016 position paper [3] as cancer-therapeutics-related cardiac dysfunction (CTRCD) and estimated using an LVEF absolute reduction of ≥10% to a value below the lower limit of normal (<53%).

Hospitalizations and all-cause mortality were extracted from computerized patient charts and the Population Registry Bureau.

### 2.3. Echocardiography

Follow-up echocardiographic examinations were performed using the same strict protocol, personnel, and equipment (General Electric (GE) system, model Vivid S70). Overall, there were two technicians included in the study; however, the vast majority of the echocardiography tests were performed by the same single technician. All echocardiography exams were signed by the same single interpreting physician. Routine LV echocardiographic parameters included LV diameters and LVEF [22]. LVEF was evaluated using 2D echocardiography and was assessed first visually and then measured using the Simpson method when a wall abnormality was observed. LV diastolic function was evaluated and classified as recommended [23,24]. RV function was evaluated using the tricuspid annular plane systolic excursion (TAPSE) [25]. In the presence of mitral valve regurgitation (MR), we estimated its severity using the ratio of the regurgitant jet to the left atrium and by measuring the vena contracta width (VCW) in the apical long axis view. [26]. VCW < 3 mm is considered mild, while VCW > 7 mm is considered severe [26]. A comparison with previous echocardiographic examinations was performed to assess changes in valvular function.

LVGLS was measured using EchoPac STE software and tracking within an approximately 5 mm-wide regions of interest (ROI). An end-systolic frame was used to initialize the LV boundaries, which were then automatically tracked throughout the cardiac cycle. Manual corrections were performed to optimize the boundary tracking as needed. Optimization of the images for endocardial visualization through the adjustment of gain, compress, and time-gain compensation controls was done prior to acquisition.

### 2.4. Statistical Analysis

Continuous variables were presented as mean (±standard deviation) or as median (interquartile range) where appropriate, depending on the distribution. Categorical variables were described as absolute numbers and percentages. Linear mixed-effect models with repeated measures were used to evaluate the temporal trends between examinations for continuous echocardiographic parameters. For categorical variables, a linear-by-linear associations statistic (Mantel–Haenszel test) was used. Independent predictors for the deterioration of mitral incompetence (defined as the occurrence of new mitral regurgitation or worsening of pre-existing mitral regurgitation) were determined in a multivariate binary logistic regression model including clinically relevant medical (age, hypertension, diabetes mellitus, hypercholesterolemia, tobacco abuse) and therapeutic (concomitant paclitaxel therapy, concomitant trastuzumab/pertuzumab therapy, concomitant left breast radiation) variables. A two-tailed *p*-value < 0.05 was considered significant for all analyses. All analyses were performed with the SPSS software (SPSS Inc., Chicago, IL, USA).

## 3. Results

### 3.1. Baseline Characteristics

From July 2016 to February 2021, 141 women diagnosed with breast cancer who received doxorubicin therapy were included, with a mean age of 51 ± 12 years. Baseline cardiac risk factors were relatively uncommon and cardiovascular disease history was rare, as displayed in Table 1.

### 3.2. Cancer Therapies

At T3, almost all patients (99%) had completed four cycles of doxorubicin therapy (a cumulative dose of 240 mg/m^2^). The potentially cardiotoxic agent trastuzumab (which is a humanized anti-human epidermal growth factor receptor 2 (HER2) monoclonal antibody) was administered to 33 (23%) patients. Other concomitant anti-cancer therapy exposures, including cyclophosphamide, paclitaxel, carboplatin, and left breast radiation, were given to 100%, 92%, 9.2%, and 28% of patients, respectively.

### 3.3. Echocardiographic Examinations

All patients underwent echocardiography evaluation at T1 and T2, with a median of 10 (6–21) and 47 (33–65) days following the first doxorubicin exposure, respectively. T3 and T4 exam results were acquired from 122 and 92 of the patients, respectively, with a median of 146 (130–208) and 245 (214–313) days following the initial doxorubicin exposure. Overall, T4 was performed at a median of 6.8 (5.7–9.0) months after the completion of the doxorubicin therapy.

### 3.4. Echocardiographic Parameters

**Figure 1:**
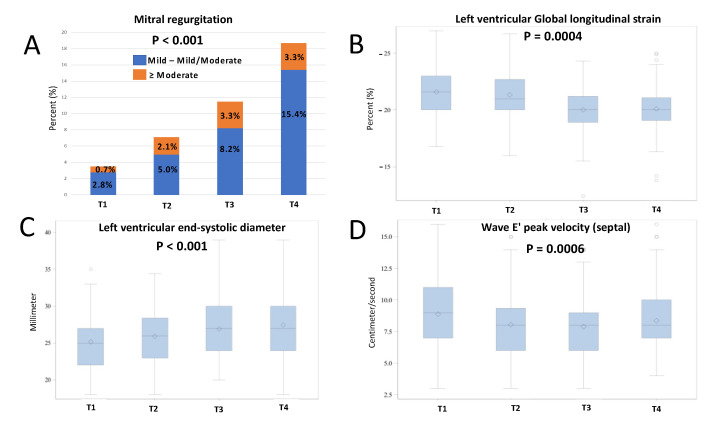
Changes in left ventricle parameters parallel to the increasing development of mitral regurgitation (**A**) The increasing development of mitral regurgitation during follow-up, in the presence of gradual left ventricle changes, including (**B**) reduction in left ventricular global longitudinal strain, (**C**) increase in left ventricular end-systolic diameter and (**D**) decrease in e’ septal values.

**Figure 2:**
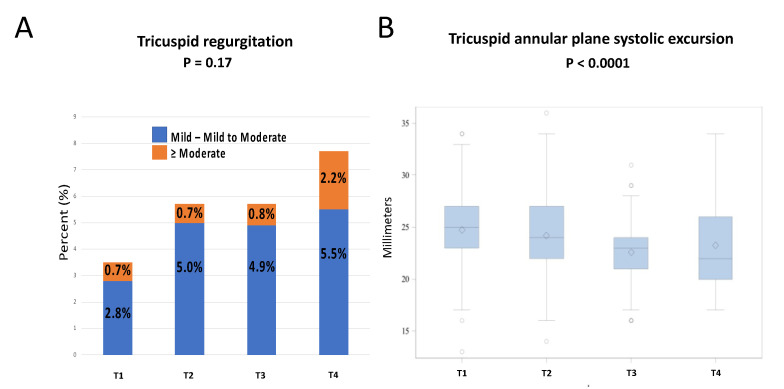
Caption Changes in right ventricle parameter parallel to the increasing development of tricuspid regurgitation (**A**) The increasing development of tricuspid regurgitation during follow-up, in the presence of reduction in (**B**) tricuspid annular plane systolic excursion values.

When evaluating the changes in LV function from T1 to T4, we observed a gradual significant statistical deterioration in LVEF (60.2 ± 1.5% to 59.2 ± 2.7%, *p* = 0.0004) and LVGLS (−21.6 (−20.0–−23.0)% to −20.0 (−19.1–−21.1)%, *p* < 0.0001), as well as an increase in the LV end-systolic diameter (LVESD) (25 (22–27) mm to 27 (24–30) mm, *p* < 0.0001) (Table 2, Figure 1). Overall, during the follow-up, a significant absolute LVEF reduction of ≥10% was observed in six patients (*p* = 0.002), of which only four patients presented with LVEF <53% (Table 2). A similar pattern was observed when evaluating the RV function, as evidenced by an overall decrease as assessed by the decline in the measurement of TAPSE (25 (23–27) mm to 22 (20–26) mm, *p* < 0.0001) (Table 2, Figure 2). While significant temporal changes were observed, the values remained within the normal range.

While significant changes were also observed in the diastolic function parameters (decrease in E/A, *p* = 0.02, and e’ septal, *p* = 0.0006), these changes mainly occurred in the time interval from T1 to T3, with a recovery or stabilization in T4. These changes did not result in a statistically significant trend in the composite classification of diastolic dysfunction grade (*p* = 0.23) (Table 2).

### 3.5. Valvular Parameters

A significant increase in the incidence of the new development of MR of any severity was observed (4% to 19%, *p* < 0.0001). Following exposure to ANT, an increasing correlative trend was seen in the portion of patients with moderate MR and above (1% to 3%, *p* = 0.13) (Table 3). Furthermore, this trend was found to be even stronger in patients treated with concomitant trastuzumab therapy (6% to 31%, *p* = 0.003, for all MR) (Table 3). In all four timepoints examinations, the mean VCW was above 3 mm, which is compatible with moderate MR (Table 4).

Similar trends were observed for tricuspid regurgitation (TR) valvular pathologies without reaching statistical significance (4% to 8%, *p* = 0.19, for TR of any severity). No cases of mitral or aortic stenosis were documented and no correlation was seen for aortic regurgitation (AR) (*p* = 0.57) (Table 3).

### 3.6. Predictors of Deterioration of Mitral Function

In a multivariate binary logistic regression model, only concomitant trastuzumab therapy (OR 5.99, 95%CI 1.35–26.6, *p* = 0.02) predicted new MR development independently, while age, hypertension, diabetes mellitus, hypercholesterolemia, smoking, paclitaxel therapy, left breast radiation, concomitant heart failure medications, or echocardiographic parameters at T1 did not (Table 5).

### 3.7. Cardiotoxicity and Cardiovascular Outcomes

During the follow-up, CTRCD was identified in four patients. Overall, two patients were admitted due to elevated troponin and chest pain, without LV dysfunction or coronary disease, and only one patient developed acute heart failure with reduced LVEF, which was considered to be tachycardia induced. Overall, three patients died during follow-up, from non-CV etiology.

## 4. Discussion

In the present study, we evaluated the incidence of new valvular dysfunction development among female patients diagnosed with breast cancer who were treated with ANT therapy. We observed a significant increase in the development of new MR, which was seen in the presence of normal LV function and regardless of exposure to left side radiation or trastuzumab therapy.

The development of valvular heart disease in cancer patients has long been considered to be solely caused by fibrosis and calcifications that are secondary to radiotherapy [3]. Only a limited number of studies [27,28,29,30] reported an increased risk of valvular dysfunction following ANT therapy, which mainly occurred in lymphoma survivors who typically also received concomitant radiation therapy. Lange et al. [31] reported a significant increase in the degree of insufficiency of MR among 27 breast cancer patients treated with ANT, followed by trastuzumab therapy.

The pathophysiology behind the development of MR following ANT is unclear. On one hand, it may develop as a secondary mechanism that is seen as a result of an overall reduction in LV function, which leads to LV dilation with structurally normal leaflets and chords [32]. The exact mechanism of cardiotoxicity produced by ANT is complex, but its histopathology is characterized by myocardial damage secondary to apoptosis, proteolysis, necrosis, and fibrosis, which deteriorates the myocardium’s ability to contract [33]. This theory was supported by the findings in our study, which showed mild yet consistent and statistically significant deterioration in LV function (both LVEF and LVGLS) and a subsequent increase in LV dimensions (LVESD) over time following ANT therapy. On the other hand, while significant changes in absolute LV function were seen, the individual LV function parameters remained mostly within the normal value range, suggesting that an additional primary mechanism was responsible for the development or deterioration of MR. This theory suggests that ANT has a direct cardiotoxic effect on the papillary muscles, causing papillary contractile dysfunction, leading to incomplete leaflet closure, and thus, the development of MR, even in the absence of mitral annular dilation [27,34,35].

Separately, trastuzumab was independently seen to cause direct cardiotoxicity, resulting in a reduction in LVEF and the development of heart failure, with an increased incidence when given concurrently with ANT [3]. Lange et al. [36] showed that among 42 breast cancer patients, trastuzumab therapy led to measurable alterations in LV and left atrium volume and an increase in the severity of the mitral valve insufficiency. In our study, trastuzumab similarly emerged as a significant independent risk factor for the development of MR, increasing the risk by 5.6-fold. Importantly, a significant MR development was observed in the subgroup of patients without trastuzumab therapy, supporting the role of ANT as a risk factor independently of trastuzumab.

Little is known about the development of TR following ANT therapy. According to Lange et al. [32], the degree of TR did not change and was not related to trastuzumab therapy. We observed a trend toward an increase in the development of TR and a significant reduction in the TAPSE value while remaining within the normal value range.

As the progression and development of cardiac dysfunction and heart failure in cancer patients are associated with increased mortality [37], the need for vigilant monitoring and early diagnosis is crucial, as it allows for the intervention by physicians who can initiate cardioprotective therapy and adjust protocolized chemotherapy regimens. The detection of incipient MR may serve as an early subclinical sign of future LV dysfunction development.

Our study had several limitations. First, it was a single-center study; however, its strength was the prospective nature following a homogenous population and the unity of all echocardiographs being performed by the same vendor, technician, and interpreting cardiologist. Second, we acknowledge that the relatively small number of patients reduced the statistical power of our results and larger trials are needed. Last, due to the short follow-up period and the low incidence of cardiac outcomes and mortality, the association of MR development to clinical outcomes was not evaluated. Long-term follow-up is needed to help assess MR development as an independent risk factor for LV dysfunction and cardiotoxicity.

In conclusion, this novel study was one of the first to focus on the development of valvular heart disease related directly to ANT therapy in breast cancer patients. A significant increase in the development of MR was observed, thus suggesting a relationship to subclinical early changes in LV functionality and a need for closer valvular evaluation and monitoring throughout the continued follow-up of cancer patients.

## Figures and Tables

**Table 1:** Baseline characteristics.

Baseline Data	
Age (years), mean ± SD	51 ± 12
Weight (kg), median (IQR)	66 (57–76)
Height (cm), mean ± SD	163 ± 7
BSA (m^2^), median (IQR)	1.72 (1.62–1.86)
Medical History	
Hypertension, *n* (%)	25 (18)
Diabetes mellitus, *n* (%)	16 (11)
Hyperlipidemia, *n* (%)	19 (14)
Smoking (current or past), *n* (%)	37 (26)
COPD, *n* (%)	1 (1)
Atrial fibrillation, *n* (%)	3 (2)
Coronary artery disease, *n* (%)	2 (1)
CVA/TIA, *n* (%)	4 (3)
Chemotherapy	
Cyclophosphamide, *n* (%)	141 (100)
Paclitaxel, *n* (%)	129 (92)
Carboplatin, *n* (%)	13 (9)
Trastuzumab/pertuzumab, *n* (%)	33 (23)
Left breast radiation, *n* (%)	39 (28)
Medications	
ACEI/ARB, *n* (%)	21 (15)
Beta blockers, *n* (%)	11 (8)
Statins, *n* (%)	20 (14)
Anti-aggregation, *n* (%)	10 (7)

BSA—body surface area; COPD—chronic obstructive pulmonary disease; CVA—cerebrovascular accident; TIA—transient ischemic attack; ACEI—angiotensin-converting enzyme inhibitor; ARB—angiotensin receptor blocker.

**Table 2:** Echocardiographic parameters.

	Time of Examination				*p*-Value
	T1 (*n* = 141)	T2 (*n* = 141)	T3 (*n* = 122)	T4 (*n* = 91)	
LV ejection fraction (%), mean ± SD	60.2 ± 1.5	59.8 ± 1.2	59.4 ± 2.1	59.2 ± 2.7	0.0004
LV ejection fraction <53%, *n* (%)	0 (0)	0 (0)	4 (3)	4 (4)	0.002
LV ejection fraction absolute decrease >10%, *n* (%)		2 (1)	6 (5)	6 (7)	0.04
LV GLS (%), median (IQR)	−21.6 (−20.0–−23.0)	−21.0 (−20.0–−22.7)	−20.0 (−18.9–−21.2)	−20.0 (−19.1–−21.1)	<0.0001
LV end-systolic diameter (mm), median (IQR)	25 (22–27)	26 (23–28)	27 (24–30)	27 (24–30)	<0.0001
LV end-diastolic diameter (mm), mean ± SD	44.2 ± 3.6	44.5 ± 3.8	44.7 ± 3.9	45.0 ± 4.0	0.36
IVS (mm), mean ± SD	9.0 ± 1.4	9.1 ± 1.9	8.9 ± 1.3	8.7 ± 1.3	0.41
LV mass (g), mean ± SD	135.7 ± 34	136.9 ± 40	135.4 ± 32	137.1 ± 36	0.82
LA volume index (mL/m^2^), mean ± SD	28.6 ± 7	29.6 ± 9	28.2 ± 8	29.5 ± 9	0.19
Diastolic dysfunction ≥ grade 1, *n* (%)	26 (19)	31 (24)	34 (28)	21 (24)	0.23
Diastolic dysfunction ≥ grade 2, *n* (%)	5 (4)	5 (4)	6 (5)	5 (6)	0.42
E/A ratio, median (IQR)	1.16 (0.96–1.41)	1.09 (0.87–1.30)	1.00 (0.83–1.34)	1.10 (0.89–1.37)	0.02
Deceleration time (ms), median (IQR)	178 (149–203)	178 (149–207)	187 (154–209)	180 (161–216)	0.43
Wave e’ peak velocity—septal (cm/s), mean ± SD	8.9 ± 2.6	8.1 ± 2.5	7.9 ± 2.5	8.4 ± 2.7	0.0006
Wave e’ peak velocity—lateral (cm/s), mean ± SD	11.0 ± 2.9	10.4 ± 3.3	10.1 ± 3.3	10.1 ± 3.2	0.05
E/e’ ratio average, median (IQR)	7.8 (6.8–10.1)	8.0 (6.7–10.1)	8.0 (6.3–10.0)	8.0 (6.0–10.8)	0.63
TAPSE (mm), median (IQR)	25 (23–27)	24 (22–27)	23 (21–24)	22 (20–26)	<0.0001
SPAP (mmHg), mean ± SD	26.4 ± 5	26.6 ± 6	24.9 ± 5	26.5 ± 6	0.05

T1, T2, T3, T4—timepoints 1–4; LV—left ventricle; GLS—global longitudinal strain; IVS—interventricular septum; LA—left atrium; TAPSE—tricuspid annular plane systolic excursion; SPAP—systolic pulmonary arterial pressure.

**Table 3:** Valvular changes during chemotherapy.

	Time of Examination				*p*-Value
	T1	T2	T3	T4	
All Patients *n* = 141	*n* = 141	*n* = 141	*n* = 122	*n* = 91	
Mitral regurgitation ≥ mild, *n* (%)	5 (4)	10 (7)	14 (12	17 (19)	**<0.0001**
Mitral regurgitation ≥ moderate, *n* (%)	1 (1)	3 (2)	4 (3)	3 (3)	0.13
Tricuspid regurgitation ≥ mild, *n* (%)	5 (4)	8 (6)	7 (6)	7 (8)	0.19
Tricuspid regurgitation ≥ moderate, *n* (%)	1 (1)	1 (1)	1 (1)	2 (2)	0.32
Aortic stenosis ≥ mild, *n* (%)	0	0	0	0	-
Mitral stenosis ≥ mild, *n* (%)	0	0	0	0	-
Aortic regurgitation ≥ mild, *n* (%)	0	3 (2)	1 (1)	1 (1)	0.57
**Concomitant trastuzumab therapy** ***n* = 33**	*n* = 33	*n* = 33	*n* = 32	*n* = 29	
Mitral regurgitation ≥ mild, *n* (%)	2 (6)	4 (12)	10 (31)	9 (31)	**0.003**
Mitral regurgitation ≥ moderate, *n* (%)	1 (3)	2 (6)	3 (9)	2 (7)	0.43
Tricuspid regurgitation ≥ mild, *n* (%)	2 (6)	2 (6)	4 (13)	4 (14)	0.21
Tricuspid regurgitation ≥ moderate, *n* (%)	1 (3)	1 (3)	0 (0)	1 (3)	0.86
Aortic regurgitation ≥ mild, *n* (%)	0	0	0	0	-
**No concomitant trastuzumab therapy** ***n* = 108**	*n* = 108	*n* = 108	*n* = 90	*n* = 62	
Mitral regurgitation ≥ mild, *n* (%)	3 (3)	6 (6)	4 (4)	8 (13)	**0.02**
Mitral regurgitation ≥ moderate, *n* (%)	0 (0)	1 (1)	1 (1)	1 (2)	0.25
Tricuspid regurgitation ≥ mild, *n* (%)	3 (3)	6 (6)	3 (3)	3 (5)	0.67
Tricuspid regurgitation ≥ moderate, *n* (%)	0 (0)	0 (0)	1 (1)	1 (2)	0.11
Aortic regurgitation ≥ mild, *n* (%)	0 (0)	3 (3)	1 (1)	1 (2)	0.51

T1, T2, T3, T4—time points 1–4.

**Table 4:** Mitral-regurgitation-related values.

	Time of Examination				*p*-Value
	T1	T2	T3	T4	
	*n* = 5	*n* = 10	*n* = 14	*n* = 17	
Vena contracta width (mm) (apical view), mean ± SD	4.5 ± 1.7	5.4 ± 2.2	4.9 ± 1.6	3.7 ± 1.6	0.18
Heart rate (beats/min), mean ± SD	72 ± 11	76 ± 9	75 ± 13	75 ± 12	0.95
Systolic blood pressure (mmHg), mean ± SD	127 ± 26	124 ± 19	130 ± 27	138 ± 19	0.67
Diastolic blood pressure (mmHg), mean ± SD	77 ± 6	71 ± 7	72 ± 14	76 ± 14	0.13

**Table 5:** Multivariate binary logistic regression for the prediction of deterioration of mitral incompetence ^+^.

Variable	OR (95%CI)	*p*-Value
Age (years)	1.02 (0.94–1.09)	0.67
Hypertension	0.51 (0.03–7.36)	0.62
Diabetes mellitus	1.22 (0.06–26.1)	0.90
Hypercholesterolemia	0.27 (0.01–6.34)	0.42
Smoking (current or history)	0.85 (0.18–4.05)	0.84
Paclitaxel therapy	0.20 (0.03–1.61)	0.13
Trastuzumab/pertuzumab therapy	5.99 (1.35–26.6)	0.02
Left breast radiation	3.98 (0.77–20.5)	0.09
ACE-I/ARB/BB therapy (at T1)	0.93 (0.06–15.7)	0.96
Left ventricular global longitudinal strain (at T1)	1.19 (0.86–1.65)	0.28
Left ventricular end-diastolic diameter (at T1) (mm)	1.00 (0.80–1.29)	0.99
Interventricular septum (at T1) (mm)	0.96 (0.47–1.96)	0.91
Diastolic dysfunction (≥grade 1) (at T1)	1.01 (0.13–7.71)	0.98

ARB—angiotensin II receptor blockers, BB—beta blockers, ACE-I—angiotensin-converting enzyme inhibitors. ^+^ Defined as new mitral regurgitation of worsening or pre-existing mitral regurgitation.

## Data Availability

Not applicable.
